# Regulation and Maintenance of an Adoptive T-Cell Dependent Memory B Cell Pool

**DOI:** 10.1371/journal.pone.0167003

**Published:** 2016-11-23

**Authors:** Marie Anson, Inês Amado, Marie-Pierre Mailhé, Emmanuel Donnadieu, Sylvie Garcia, François Huetz, Antonio A. Freitas

**Affiliations:** 1 Institut Pasteur, Départment d’Immunologie, Unité de Biologie des Populations Lymphocytaires, Paris, France; 2 CNRS, URA1961, Paris, France; 3 Institut Cochin, Inserm, U1016, Paris, France; 4 CNRS, UMR8104, Paris, France; 5 Université Paris Descartes, Sorbonne Paris Cité, Paris, France; Universita Cattolica del Sacro Cuore, ITALY

## Abstract

We investigated the ability of monoclonal B cells to restore primary and secondary T-cell dependent antibody responses in adoptive immune-deficient hosts. Priming induced B cell activation and expansion, AID expression, antibody production and the generation of IgM^+^IgG^-^ and IgM^-^IgG^+^ antigen-experienced B-cell subsets that persisted in the lymphopenic environment by cell division. Upon secondary transfer and recall the IgM^-^IgG^+^ cells responded by the production of antigen-specific IgG while the IgM^+^ memory cells secreted mainly IgM and little IgG, but generated new B cells expressing germinal center markers. The recall responses were more efficient if the antigenic boost was delayed suggesting that a period of adaptation is necessary before the transferred cells are able to respond. Overall these findings indicate that reconstitution of a functional and complete memory pool requires transfer of all different antigen-experienced B cell subsets. We also found that the size of the memory B cell pool did not rely on the number of the responding naïve B cells, suggesting autonomous homeostatic controls for naïve and memory B cells. By reconstituting a stable memory B cell pool in immune-deficient hosts using a monoclonal high-affinity B cell population we demonstrate the potential value of B cell adoptive immunotherapy.

## Introduction

Immune responses to infectious agents have different out-comes that can either protect or fail to control disease. Protection from re-infection relies on the establishment of efficient secondary immune responses that require the generation of antigen-specific “memory” B and T lymphocytes. The generation and selection of T-cell dependent “memory” B cells involves distinct molecular mechanisms: immunoglobulin isotype recombination and somatic hyper mutation, both dependent on the expression of AID [1]. Therefore, a long-standing paradigm defined memory B cells as IgM^-^IgG^+^ isotype switched cells [2]. Different lines of evidence indicate that this is not always the case. In humans, it has been shown that some IgM^+^ B cells bear the phenotype of other memory cells, being CD27^+^, and carry frequent point mutations in the V region of the Ig genes, suggesting that they must represent highly selected B cell populations [3]. In mice, populations of CD19^+^IgM^+^ able to mount secondary responses have been identified [4–7]. Overall these findings suggest that the T-cell dependent memory B cell pool comprises distinct subsets of memory B cells with different properties and effector functions [4–6].

The biological properties that ensure the long-term persistence of memory and efficient secondary antibody responses have not been yet completely established. While initial studies proposed that after transfer memory B cells faded rapidly [8, 9] suggesting that long-lasting memory required the continuous recruitment of new cells [8] and/or antigen persistence [9, 10], others suggested that memory B cells were able of extended survival without cell division [11] in the absence of antigen [2]. Long-term persistence of antibody responses has also been attributed to populations of long-lived plasma cells mainly resident in the bone marrow following immunization [12, 13]. The demonstration of the compartmentalization of “antibody memory” into different cellular layers suggested that the separate subsets of memory B cells behave differently. Accordingly, it has been reported that IgG^+^ cells that could rapidly respond upon challenge did not persist long, while IgM^+^ cells could generate a second wave of germinal center responses allowing persistence of memory [4–6, 14].

Currently, immunotherapy approaches using passive antibody transfer [15, 16]) is limited by the short half-life of immunoglobulin. Therefore new therapy strategies may require the adoptive transfer of high-affinity memory B cells, ready to respond and able to persist. The development of these new strategies requires a profound understanding of the mechanisms that regulate memory B cell numbers and ensure long persistence upon adoptive transfer. Moreover, knowledge of the mechanisms that determine the size of the memory B cell pool may be also critical to device new reconstitution strategies. So far, studies comparing populations of naïve and memory B cells have been hindered both by the vast clonal heterogeneity of the cells involved and by our inability to generate significant numbers of antigen specific memory B cells. Indeed in a normal laboratory mouse the population of B cells bearing a “memory IgG^+^ phenotype’ represent a small fraction of the total B cell pool (<0.5%) and upon immunization the number of the clonal diverse antigen-specific memory B cells generated is generally very limited (<10^3^) [1, 6].

To circumvent these limits, we decided to compare the properties of homogeneous populations of naïve and memory B cells of known antigen specificity, belonging to the same clone. We used SW_HEL_ transgenic mice where B cells bear a high-affinity BCR specific for HEL and are capable of class switch recombination and somatic hypermutation (SHM) [17, 18]. To identify “memory B cells” the SW_HEL_ mice were crossed with mice where AID transcription provokes the permanent expression of an YFP reporter in post-germinal center lymphocytes [19]. These mice were in a Rag2-deficient background and therefore contain a pure population of monoclonal HEL-specific B cells. To generate memory cells, purified naïve B cells from the SW_HEL_.AID/YFP.Rag2^-/-^ mice were transferred into adoptive hosts together with monoclonal OVA-specific CD4^+^ T cells from OTII.Rag2^-/-^ TCR transgenic mice. Upon immunization with OVA-HEL complexes, we obtained a significant number of persisting HEL-specific IgM^+^IgG^-^YFP^+^ and IgM^-^IgG^+^YFP^+^ memory B cells, number that did not correlate to the number of precursor naïve cells initially injected suggesting that the memory B cell pool is regulated independently. We characterized the functional capacity of these two memory cell types in immune deficient hosts.

## Materials and Methods

### Mice

B6 and B6.Rag2-/- [20] mice were kept at the Centre Des Techniques Avancées (CDTA), Centre National de la Recherche Scientifique (CNRS), Orleans, France; SWHEL.AID/YFP.Rag-/- mice, obtained by crossing SWHEL (18)(a gift of Dr. Robert Brink) and AID/YFP [19](a gift of Dr. Rafael Casellas) with B6.Rag2-/- mice. OTII.Rag-/- mice were kept in our animal facilities at the Pasteur Institute. Experiments were preformed according to Pasteur Institute Safety Committee in accordance with French and European guidelines and the ethics Committee of Paris 1 (permits 2010–0002, -0003 and -0004). Euthanasia of the mice was performed by cervical dislocation. This specific study was approved by the European Research Council (ERC) committee related to the grant AdG09 249740-QSIS. The general status of the mice was controlled daily by monitoring the appearence of obvious pain, distress or suffering (prostration, respiratory issues, loss of weight). The end-point of the experiment was determined by a loss of more than 20% of the weight or as soon as the distress signs appeared. In this case, experiment was stopped and the animals were euthanized.

### Adoptive transfer for the generation of memory B cells

Single-cell suspensions of B cells from spleens and lymph nodes of SW_HEL_.AID/YFP.Rag^-/-^ mice together with CD4^+^ T cells from spleens and lymph nodes of OTII.Rag^-/-^ mice were transferred intravenously into the retro-orbital sinus of B6.Ly5^a^IgH^a^ or B6.Rag2^-/-^ recipient mice. Mice received 10^6^ HEL^+^ B cells and 10^6^ CD4^+^ T cells unless stated otherwise. Mice were immunized 24H later with 1 mg of Ovalbumin coupled to Hen Egg Lysozyme (OVA-HEL) in 50μg of Alu-S-Gel (Serva) we determined as the optimal dose of Ag (data not shown).

### Adoptive cell transfer for secondary responses

Naive cells from SW_HEL_.AID/YFP.Rag^-/-^ mice and memory B cells subsets from immunized B6.Rag^-/-^ hosts mice were purified from spleens and lymph nodes by flow cytometry sorting. Single-cell suspensions containing 5×10^4^ B cells and 10^6^ T cells were transferred intravenously into B6.Rag2^-/-^ recipient hosts. The purity of sorted cells was above 98%. 24 h after transfer, mice were immunized with 1 mg of OVA-HEL.

### Flow cytometry and cell sorting

Spleen, bone marrow, inguinal and mesenteric lymph nodes single-cell suspensions were stained for cell surface or intracellular proteins with appropriate combinations of the following monoclonal antibodies conjugated to pacific blue, Qdot-655, Brillant Violet 605, allophycocyanin, peridinin chlorophyll protein–cyanine 5.5, phycoerythrin, phycoerythrin-cyanine7: anti-CD19 (6D5), anti-IgM (R6-60.2), anti-IgG1 (X56), anti-CD138 (281–2), anti-Gl7 (Gl7), anti-CD95 (Jo2), anti-CD62L (MEL-14), anti-CD69 (H1-2F3), anti-BAFFR (7H22-E16), anti-CXCR5 (L138D7), anti-IA^b^ (AF6-120.1), anti-CD80 (16-10A1), anti-CD73 (TY-11-8) and anti-PDL2 (TY25) and anti-Ki-67 (mm1) purchased from Becton Dickinson Pharmingen, Biolegend, Invitrogen and eBioscience. Cells were also stained with HEL (Sigma) coupled with AF594 using Alexa Fluor® 594 Protein Labeling Kit from Life technologies. Before staining, cells were treated with Fc-Block (CD16/CD32, Becton Dickinson Pharmingen). Dead cells were excluded during analysis according to their light-scattering characteristics. For intracellular stainings, cells were first stained with antibodies specific for cell surface antigens. Then, cells were fixed and permeabilized according the manufacturer’s recommendations (BD Bisciences). For proliferation assay, mice were injected i.p. with 50 mg/kg of BrdU (Sigma-Aldrich) and were killed 24 or 72 hours later. Incorporated BrdU was detected intracellularly using anti-BrdU APC-conjugated antibodies according to the manufacturer’s recommendations (BD Biosciences). All data acquisitions and analyses were performed with LSRFortessa (Becton Dickinson) interfaced with BD FACSDiva (Becton Dickinson) and FlowJo (Tree Star) software. Subsets of memory B cells were sorted as CD19^+^HEL^+^YFP^+^IgM^+^ or IgG^+^ and naive cells as CD19^+^HEL^+^YFP^-^IgM^+^ using a FACSAriaIII flow cytometer. The purity of the sorted populations varied from 90–95%.

### ELISA and ELISpot assay

Sera HEL-specific Ig concentrations were quantified by ELISA. Plates were coated with HEL and saturated with PBS-5% Milk. Dilutions of sera were added. After incubation (2 hours, 37°C) and washing, HRP-labeled anti-mouse IgM or IgG antibodies were added. After incubation and washing, bound antibodies were revealed with the substrate O-phenylenediamine and H2O2. The reaction was stopped after 10 min. by addition of 10% SDS and the absorbance read at 492nm in a multiscan spectrometer. Ig concentrations were determined by comparing the displacement of the dilution curves in the linear interval between standards at a concentration of 1 mg/ml and the serum samples.

The quantification of IgG or IgM secreting cells was assayed by ELISpot technique. Briefly, plates were coated with HEL. After saturating, the cells were distributed into the micro wells in RPMI1640-2%FCS. The plates were incubated for 12 h at 37°C, 5% CO2 atmosphere. After extensive wash, plates were incubated with goat anti-mouse IgM or anti-IgG labeled with alkaline phosphatase. After washing, the revealing substrate was added (2,3 mM 5-bromo-4-chloro-3-indolyl phosphate diluted in 2-amino-2-methyl-1-proprenolol buffer).

### Confocal microscopy

Spleens from 14 day-immunized mice were initially fixed with paraformaldehyde and embedded in 4% low-gelling-temperature agarose (type VII-A; Sigma-Aldrich) prepared in PBS. 150-μm slices were cut with a vibratome (VT 1000S; Leica) in a bath of ice-cold PBS. For immunolabeling, samples were saturated with PBS supplemented with 10% of fetal calf serum, then were labeled with primary antibodies anti-B220-APC (clone RA3-6B2) and anti-IgD-PE (clone 11-26c.2a) and analyzed with a spinning disk confocal microscope equipped with a CoolSnap HQ2 camera (Photometrics) and a 20x objective. Images were acquired and analyzed with MetaMorph 7 imaging software Molecular Devices).

### Statistical analysis

Sample means were compared using the Student’s *t* test. Sample means were considered significantly different at *p* < 0.05.

## Results

### Different types of memory B cell subsets upon adoptive transfer of monoclonal naïve B cells

**Fig 1:**
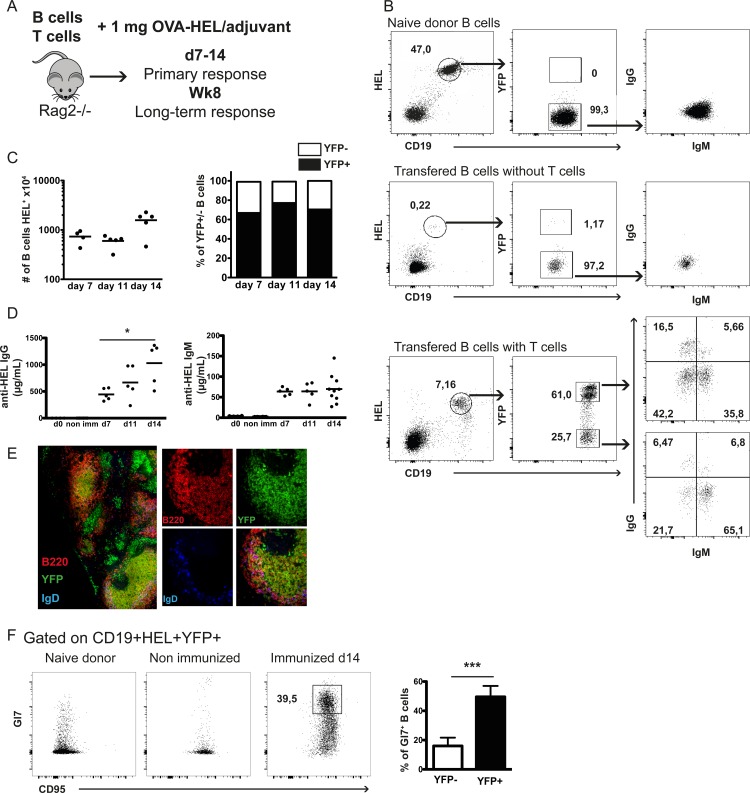
Development of B cell primary response upon adoptive transfer of SW_HEL_.AID/YFP.Rag2^-/-^ B cells. (A) Rag2-/- hosts were injected intravenously with naive SW_HEL_.AID/YFP.Rag2^-/-^ B cells together with OTII.Rag2^-/-^ naive CD4 T cells. Recipient mice were immunized 24 hours later and B cell response analyzed 7, 14 days and 8 weeks after. (B) Flow cytometric analyses of specific HEL^+^ CD19^+^ (left panels) splenic B cells from SW_HEL_.AID/YFP.Rag2^-/-^ naive donor (upper panels), immunized mice reconstituted only with B cells (middle panels) and immunized mice reconstituted with both B and T cells 14 days after immunization (lower panels), for the expression of YFP (middle panels), and IgM and IgG (right panels). (C) Number of splenic B cells recovered from the recipient mice (left panel) and repartition of AID/YFP- and AID/YFP+ among splenic B cells (right panel) 7, 11 and 14 days after immunization. (D) Seric levels of anti-HEL specific IgG (left panel) and IgM (right panel) in immunized recipients 7, 11 and 14 days after immunization. (E) AID/YFP (green), B220 (red), IgD (blue) (right panels) and merged **(**left and right panels) expression by confocal microscopy analysis of spleen slices 14 days after immunization. (F) Analysis of germinal center B cells in immunized mice. Left panel shows the co-expression of Gl7 and CD95 by B cells from naive (left dot plot), non-immunized (middle dot plot) and 14 day-immunized mice (right dot plot). Right panel shows the % of Gl7hi CD95+ cells among AID/YFP- (white bar) and AID/YFP+ (black bar) splenic B cell subsets from 14 day-immunized mice. Data (mean ± SEM) are shown for one experiment representative of 3, with 4–5 mice per group. Significances were calculated using Student *t*-tests, *, P<0.05; ***, P<0.001.

During an immune response the complexity of determinants expressed by immunizing antigen and the degeneracy of antigen-specific recognition results in a vast heterogeneity of responding cells rendering impossible the direct comparison of the properties of naïve and memory B cells belonging to the same clone. We have devised an experimental system that permits the comparison between naïve and memory B cells expressing the same antigen receptor and allows marking permanently memory B cells. For that purpose we used SW_HEL_ transgenic mice in a Rag2-deficient background holding a single population of monoclonal B cells, all bearing a high-affinity BCR specific for HEL and capable of class switch recombination and somatic hypermutation (SHM) [17, 18]. To identify antigen-experienced B cells the SW_HEL_.Rag2^-/-^ mice were crossed with mice where AID transcription induces the permanent expression of an YFP reporter in post-germinal center lymphocytes [19]. Since in intact Tg mice immune responses were not traceable, probably because of the presence of low level pre-existing anti-HEL antibodies that neutralize the immunizing protein, we used an adoptive cell transfer strategy to study the ability of the high affinity monoclonal B cell to reconstitute response in immune-deficient hosts and generate antibody memory. Purified naïve B cells from the SW_HEL_.AID/YFP.Rag2^-/-^ mice were transferred into Rag2-deficient mice together with monoclonal OVA-specific CD4^+^ T helper cells from OTII.Rag2^-/-^ TCR transgenic mice. The day after, host mice were immunized with OVA-HEL complexes (Fig 1A). In these conditions, antigenic challenge resulted in B cell activation and the development of significant numbers of CD19^+^HEL^+^AID/YFP^+^ B cells, which were not detected in non-immunized mice or in mice immunized in absence of helper T cells (Fig 1B). We followed the early kinetics of this response. The number of HEL-specific B cells increased from the initial 2x10^6^ transferred to about 15x10^6^ at day 14 (Fig 1C left) the B cells expressing AID/YFP being the dominant population (Fig 1C right). A fraction of the HEL-specific B cells underwent class switch recombination and at day 14 we recovered both IgM^+^IgG^-^AID/YFP^+^ and IgM^-^IgG^+^AID/YFP^+^ cell populations (Fig 1B). B cell expansion and phenotypic changes were accompanied by the production of IgM and IgG HEL-specific antibodies (Fig 1D). Two weeks after antigenic challenge we observed the formation of germinal centers in the spleen of the host mice (Fig 1E). Coherently we found that while upon adoptive transfer all B cells expressed CD95, only after antigenic challenge most YFP^+^ B cells expressed the germinal center specific marker GL7 (Fig 1F). In conclusion, the adoptive cell transfer strategy allowed the development of a primary immune response with B cell activation and expansion, induction of AID expression, class switch recombination, antigen-specific IgM and IgG antibody production and germinal center formation.

### Kinetics of memory B cell generation

**Fig 2:**
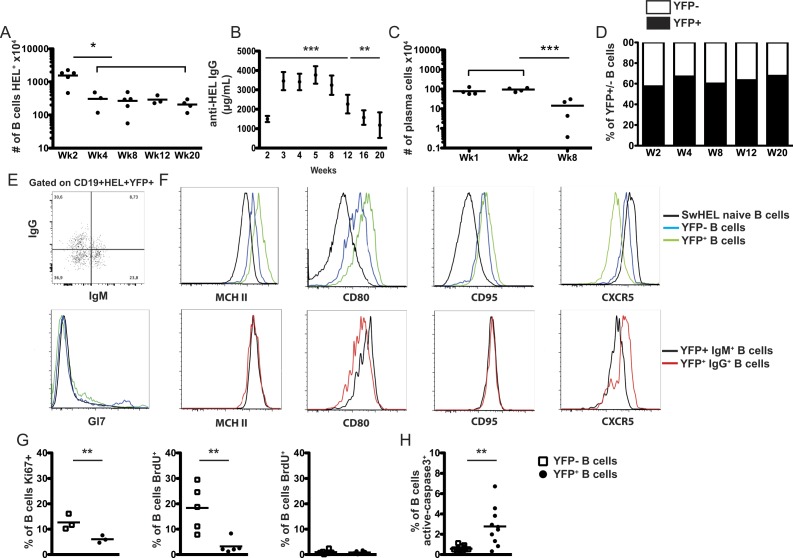
Long-term maintenance of memory B cells. Rag2-/- recipient mice were injected with naive SW_HEL_.AID/YFP.Rag2^-/-^ B cells and OTII.Rag2-/- naive CD4 T cells and immunized as in Fig 1. Mice were analyzed at 2, 4, 8, 12 and 20 weeks after immunization. (A) Number of splenic B cells recovered. (B) Seric level of anti-HEL specific IgG1. (C) Number of splenic CD138+ plasma cells determined by flow cytometry. (D) Repartition of splenic AID/YFP+ (black bars) and AID/YFP- (white bars) B cells. (E) Flow cytometric analyses of IgG and IgM expression by AID/YFP+ B cells. (F) Flow cytometric analyses of MHC II, CD80, CD95, CXCR5 and Gl7 expression by splenic AID/YFP+ (green lines), AID/YFP- (blue lines) and naive (black lines, upper histograms), and IgM+ (black lines) and IgG+ (red lines, lower histograms) B cells from naive, and eight-week immunized mice. (G) The % of splenic proliferating B cell was assessed 8 weeks after immunization by flow cytometric analysis of Ki67 expression (left) and BrdU incorporation 24h (middle) or 72h (right) after BrdU injection. (H) The % of apoptotic splenic B cell was assessed by flow cytometric analysis of active caspase 3 level 8 weeks after immunization. Data (mean ± SEM) are shown for one experiment representative of 2, with 4–5 mice per group. Significances were calculated using Student *t*-tests, *, P<0.05; ***, P<0.001.

**Fig 3:**
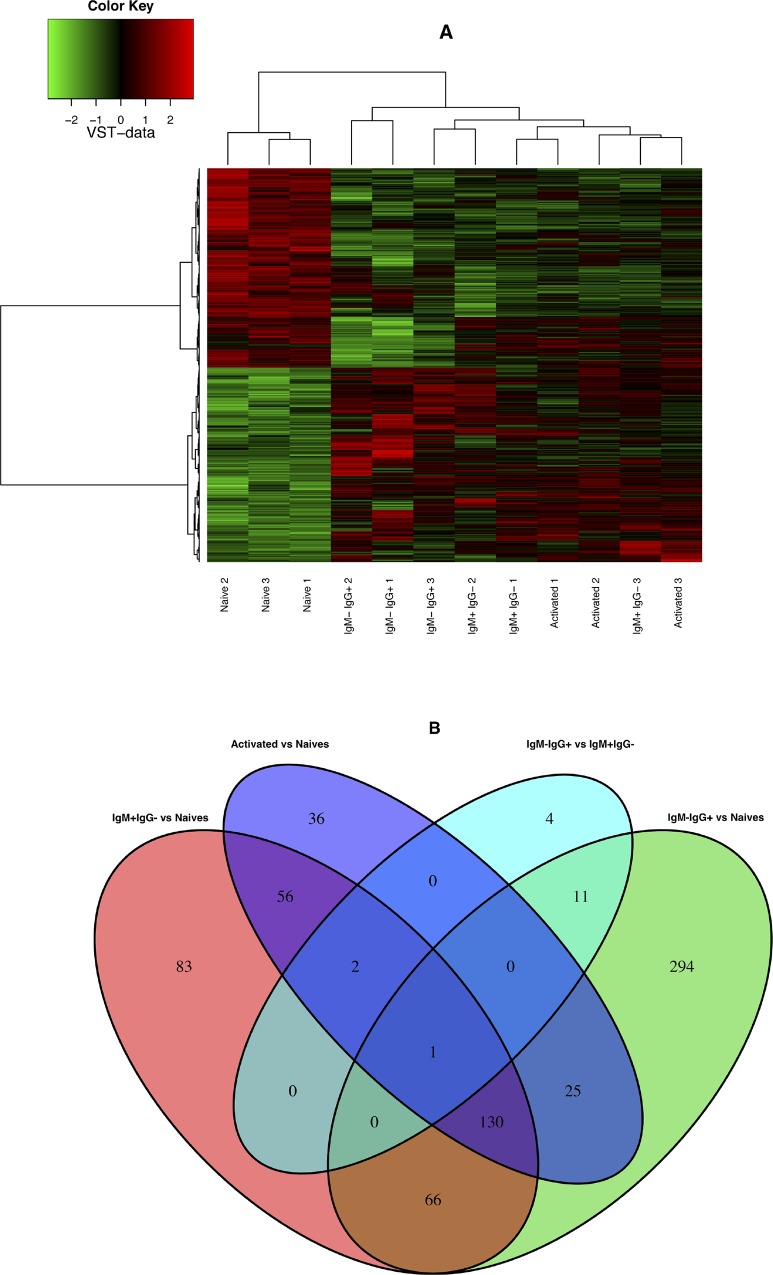
Transcriptional program of memory B cell subsets.

We studied the evolution of the B cell response. From two weeks onwards the total number of B cells contracted and at four weeks we recovered about 2-4x10^6^ cells, number that remained stable up to week 20 (Fig 2A). High titers of HEL-specific IgG were kept from week 3 to 8, declined thereafter, but were still significantly elevated 20 weeks later (Fig 2B). A population of cells secreting HEL-specific Igs was present in the spleen (Fig 2C), but not in the BM (not shown) even at the late time points. About 60% of the recovered cells exhibited the phenotype of antigen-experienced (“memory”) CD19^+^HEL^+^AID/YFP^+^ expressing either IgM or IgG (Fig 2D and 2E). We compared the phenotype of the two AID/YFP^+^IgM^+^ and AID/YFP^+^IgM^-^IgG^+^ memory cell populations recovered with that of the naïve B cells (Fig 2F). We found that antigen-experience and naïve B cells expressed similar levels of CD62L, CD69 and BAFFR (not shown). Antigen-experienced cells presented sustained expression of CD95 and increased levels of PNA, but the vast majority lost expression of the germinal center marker GL7 present at earlier times post-immunization (Fig 2F compare to Fig 1F). Compared to naïve B cells, AID/YFP^+^ cells expressed higher levels of CD80 and MHC class II and down-regulated expression of CXCR5 (Fig 2F). These findings indicate that the post-germinal center AID/YFP^+^ B cells express an activated phenotype [5, 21], have increased antigen-presenting capacity [22], but may loose the ability to re-enter primary follicles being CXCR5^low^ [23]. We have also compared the patterns of gene expression (RNAseq) by naïve, activated (YFP^-^ cells of immunized mice) and both populations of YFP^+^ memory cells. The data shows a clear discrimination of naïve and activated/memory cells while indicating only minor differences between both subsets of YFP^+^ memory cells (Fig 3).

mRNA was isolated from sort-purified Naïve (CD19^+^HEL^+^YFP^-^IgM^+^) IgM^+^IgG^-^ or IgM^-^IgG^+^HEL^+^ CD19^+^YFP^+^ memory B cells from spleen of different recipient mice. Total RNA was isolated using the RNeasy Micro Kit (QIAGEN). RNA quality and concentration were assessed using RNA 6000 Pico LabChips with a 2100 Bioanalyzer (Agilent Technologies). Total RNA (0,1 to 1 μg) was then converted into a library of template molecules of known strand origin using the TruSeq Stranded total RNA sample preparation kit (Illumina) as recommended by the manufacturer. The validated libraries were then subjected to DNA sequencing. The analysis is performed using the R software, Bioconductor packages including DESeq2 and the PF2tools package (version 1.2.9) developed at PF2 (Institut Pasteur). Normalization and differential analysis are carried out according to the DESeq2 model and package (version 1.8.1). Fig 3A shows a representative heat map of the different cells populations. Fig 3B shows the Venn diagram showing total number of differentially expressed features for each comparison. The RNA sequence data has been deposited in NCBI’s Sequence Read Archive database under accession number GSE79672 (http://www.ncbi.nlm.nih.gov/geo/query/acc.cgi?acc=GSE79672).

### Memory B cells are actively dividing cells

Late in the immune response persistent B cell numbers were kept by active cell division as a significant fraction of the cells were Ki67^+^ (Fig 2G left) and incorporated BrdU (Fig 2G middle). The frequency of BrdU^+^ cells was higher among the AID/YFP^-^ cells (15%) than in the major AID/YFP^+^ memory population (3%) and similar between the IgM^+^ and IgM^-^ AID/YFP^+^ populations (Fig 2G middle and not shown). Three days after BrdU pulse populations were clear of BrdU^+^ cells (Fig 2G right) attesting their high division rate. In spite of their increased proliferation rate, memory cells numbers were stable indicating that proliferation may be compensated by cell death as suggested by the frequency of caspase3^+^ cells (Fig 2H). The frequency of Caspase3^+^ cells was higher among the AID/YFP^+^ cells suggesting that a fraction of these cells may represent cells undergoing terminal differentiation. Importantly, these findings demonstrate that the transfer strategy allowed the generation of significant numbers of persisting antigen-experienced YFP^+^ cells.

### The number of memory B cells is independent the number of naïve B cell progenitors

**Fig 4:**
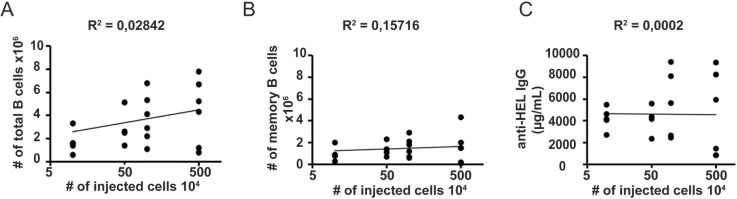
Regulation of the size of the memory B cell pool. Rag2-/- recipient mice were injected with different numbers of naive SW_HEL_.AID/YFP.Rag2^-/-^ B cells and OTII.Rag2-/- naive CD4 T cells and immunized as in Fig 1. The correlations between the number of injected B cells and (A) the number of total splenic B cells recovered, (B) the number of splenic AID/YFP+ memory B cells recovered 8 weeks after immunization and (C) the level of anti-HEL specific IgG in the serum are shown. For each plot, linear regression coefficient R2 is shown.

It is not yet known whether the number of antigen-experienced memory B cells correlated to the number of naïve B cells or if it is controlled independently of the initial number of antigen-specific B cells present. To approach this question we transferred different numbers of mature naïve B cells from SW_HEL_.AID/YFP.Rag2^-/-^ donors (ranging from 10^5^ to 5.10^6^) into Rag2-deficient mice together with an excess of CD4^+^ T helper cells (10^6^) and immunize the hosts the day after cell transfer with OVA-HEL in optimal non-limiting quantities. To directly compare the results obtained after the transfer of different all numbers we allowed the responses to reach steady-state eight weeks after antigenic challenge. We studied the amplitude of the immune response by measuring the serum titers of HEL-specific IgG antibodies and enumerating the number of HEL-specific B cells recovered. We found that in the presence of excess T cell help, the levels of the HEL-specific IgGs (Fig 4C), and both the total number of HEL-specific (Fig 4A) and of memory YFP^+^ B cells recovered (Fig 4B), did not correlate to the number of antigen specific naïve B cells initially transferred.

### Differential functionalities of both subsets of memory B cells

**Fig 5:**
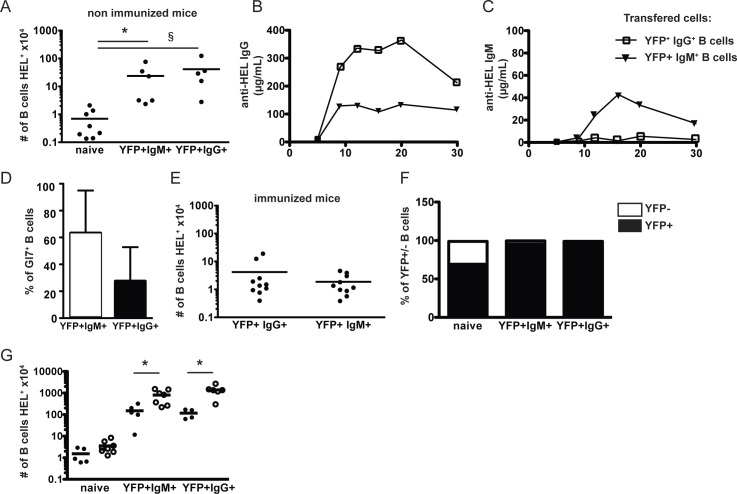
Functions of AID/YFP+ memory B cell subsets. Rag2-/- recipient mice were injected with naive SW_HEL_.AID/YFP.Rag2^-/-^ B cells and OTII.Rag2-/- naive CD4 T cells and immunized as in Fig 1. Eight weeks after immunization, AID/YFP+ IgM+ and AID/YFP+ IgG+ subsets were isolated and transferred alone into secondary Rag2-/- recipient mice. Naive B cells were transferred as controls. (A) Numbers of splenic B cells recovered from the different secondary hosts without immunization. (B) The secondary hosts were immunized 1 day after transfer and the seric levels of anti-HEL specific IgG (C) and IgM were measured 6, 9, 12, 16, 20 and 30 days after immunization. (D) the % of splenic Gl7 hi B cells in secondary hosts transferred with YFP+ IgM+ (white) or YFP+ IgG+ (black) B cells was analyzed by Flow Cytometry 3 weeks after immunization and (F) the relative % of AID/YFP- and AID/YFP+ cells was assessed. In another settings, (G) the different secondary hosts were immunized either 1 day (black circles) or 30 days (white circles) after transfer and the number of total splenic B cells recovered from the spleen of different secondary hosts was determined 6 weeks after immunization. Data (mean ± SEM) are shown for one experiment representative of 2, with 4–5 mice per group. Significances were calculated using Student *t*-tests, *, P<0.05; ***, P<0.001.

Memory B cells are defined functionally by their ability to induce secondary IgG antibody responses upon secondary antigenic challenge. We investigated whether the subsets of AID/YFP^+^IgM^+^ and AID/YFP^+^IgM^-^IgG^+^ antigen-experienced (memory) B cells persisting at late time points could mount secondary IgG responses and persist after secondary transfer. For this purpose we followed two different experimental strategies. In the first, 5x10^4^ cells of either IgM^+^ or IgM^-^IgG^+^ memory B cells, were transferred with an excess helper OTII CD4^+^ T cells into secondary Rag-deficient hosts that were boosted with OVA-HEL the day after cell transfer. In the absence of immunization antibody levels were undetectable (not shown) and three weeks after transfer recovery of both memory B cell subsets was about 10–20% of the initial cell input, exceeding naïve B cell recovery (Fig 5A), supporting the notion that memory B cells may not require specific ligand recognition to survive (2). One cannot exclude, however, that cross-reactivity of the BCR transgene with environmental antigens may allow signaling sufficient to maintain naïve and memory cell survival in the absence of HEL [24]. Following immunization, the secondarily transferred AID/YFP^+^IgM^-^IgG^+^ cells responded promptly with the exclusive production of significant levels HEL-specific IgG thus confirming their memory statute (11). The AID/YFP^+^IgM^+^ B cells in response to antigenic boost produced only limited amounts of IgM antibodies (Fig 4B), little IgG antibodies, but did generate GL7^+^ B cells more efficiently than the IgG^+^ memory B cell population (Fig 5D). Thus the IgM^+^ subset may contain precursors able to generate a secondary germinal center reaction and a new progeny of IgG^+^ effectors (4). With time antibody levels decayed rapidly suggesting that the number of transferred memory B cells declined in the secondary hosts after antigenic boost. Indeed, IgM^+^ and IgG^+^ memory B cells failed to expand and 3 weeks after immunization cell recovery was similar to the retrieval observed in the non-immunized hosts (compare Fig 5E and 5A). In similar experimental conditions, naïve B cells following immunization expanded, acquired AID/YFP expression and their numbers more than doubled the number initially injected (Figs 5F and 2A). These data suggest that a significant fraction of the memory B cells generated have a reduced expansion capacity being programmed for rapid differentiation for effector functions.

Besides long-term survival memory B cells must maintain functional activity in the absence of nominal antigen to be fully effective. To test this we used an alternative approach where memory cells were parked in secondary Rag-deficient hosts for 30 days before re-immunization. We found that under these conditions antigenic challenge resulted in the production of HEL-specific IgG antibodies and in a 100 fold increase in the number of cells recovered, expansion that largely exceed that observed after immediate challenge (Fig 5G).

## Discussion

The aim of this study was to characterize the fate of activated B cells and the generation of memory B cells. To do this, we adoptively transferred monoclonal B cells into immune deficient hosts followed by immunization in presence of T cell help. This strategy resulted in the development of different B cell memory subsets, namely IgM^+^ and IgG^+^, as described for in situ generated memory cells [4, 6, 14]. These findings indicate that distinct memory B cell subsets are not the result of the heterogeneity of initially responding naive cells, but originate from the differentiation of a single B cell clone.

While studying the respective rate of proliferation of both types of memory B cells, we found the same high rate of proliferation for IgM^+^ and IgG^+^ memory B cells. These results contrast with previous published data. First it was been reported that “in situ” memory B cells persist as resting non-dividing cells [11, 25]. However, we have shown that upon adoptive transfer and in absence of competing cells, B cells increase their division rate to occupy the available empty niche [26], which may explain the higher division rate observed here using this adoptive cell transfer strategy. Secondly, comparing life spans among heterogeneous memory B cell populations it was previously reported a lower division rate among the IgM^+^ subset compared to the IgG^+^ polyclonal subset [6]. Differences in BCR affinity between IgM^+^ and IgG^+^ memory clones may explain the higher division rate previously observed among the IgG^+^ cells [6]. In contrast we compared memory B cell subsets belonging to the same clone bearing the same high affinity BCR. Overall these observations support the notion that lymphocyte division rates and life spans are not an intrinsic cell property, but rather determined by the environment and the presence of competing populations [27]. They demonstrate that upon the correct conditions memory B cells can persist by cell division.

An important question was whether the number of memory B cells depends on the number of initial naïve B cells. We found that, in the presence of an excess of T cell help, that was not the case. However, it was previously reported during polyclonal responses that serum titers of anti-HSA was proportional to the number of cells transferred into irradiated mice [28]. It is possible that limited antigen-specific T-B cell encounters may constraint the number of responding B cells and thus determine linear precursor-progeny between naïve and memory B cells. Our findings indicate that within a single clone the number of precursor naive B cells present in the peripheral B cell pool does determine neither the intensity nor the final number of memory B cells in response to an optimal dose of antigen. They suggest that the size of memory B cell pool may be controlled independently of the number of naïve B cell precursors and that in the absence of clonal competition the memory niche can be filled with a single monoclonal population. Considering diverse polyclonal populations, the limited niche for memory cells will imply strong competition among clones resulting in the selection of best fit (high affinity) cells: rare mutated clones being able to out compete more frequent but less avid clones. In our settings, the transgenic memory B cells are likely to counter select any new mutant clones since they express a very high affinity BCR selected in the course of a secondary immune response [29]. Thus, notwithstanding the expression of AID and proliferation we did not detect any BCR VH and VL Ig-chain nucleotide mutations among the recovered memory B cells (not shown). These findings may have implication for vaccination protocols as they indicate that each new antigenic exposure or unrelated immunization would add extra competing clones supporting the need for repeated antigenic boosts to prevent memory B cell attrition. They also demonstrate that the memory B cell pool can be reconstituted from a relatively small number of antigen-specific cells.

It is likely that the relatively poor memory B cell expansion observed after immediate boost after adoptive transfer could be due to the lack in Rag-deficient hosts of the appropriate environment required for memory B cell survival and function. It should be pointed out that B cell transfer into transgenic ML5 Rag-deficient hosts expressing low levels of HEL [29] resulted in rapid cell loss and recovery suggesting that in these hosts, B cells are trapped by antigen in locations were they are unable to survive (not shown). Nevertheless, it has been shown that B cells can drive the maturation of follicular dendritic cells and the organization of lymphoid follicles [30]. Similarly, transferred helper cells may also modify their immediate environment. Thus, by allowing lymphocytes to adapt and modify their immediate environment we improved their response and more important, we recovered the memory B cell pool size present in the original donor mice.

In this study we show that it is possible to fully reconstitute a primary response and the establishment of antibody memory in immune deficient mice after adoptive transfer of antigen-specific monoclonal B cells together with a population of monoclonal helper T cells. Indeed, it is generally believed that in immune deficiencies, B cell therapy has restricted application due to intrinsic defects of host’s lymphoid organs structure that may prevent development of immune responses, germinal center formation, establishment of antibody memory and limit cell survival. In contrast we showed that after adoptive transfer in immune deficient hosts antigen immunization induced B cell activation and expansion, induction of AID expression, class switch recombination, antigen-specific IgM and IgG antibody production, germinal center formation and the generation of two subsets of AID/YFP^+^IgM^+^IgG^-^ and AID/YFP^+^IgM^-^IgG^+^ antigen-experienced B cell subsets able to persist in a lymphopenic environment by cell division mimicking responses obtained in intact non-Tg mice [4]. Upon challenge the AID/YFP^+^IgM^-^IgG^+^ cells responded promptly with the production of HEL-specific IgG while the AID/YFP^+^IgM^+^ B cells secreted only limited amounts of IgM antibodies and fail to produce IgG. In contrast the AID/YFP^+^IgM^+^ B cells could give rise to new GL7^+^ B cells, suggesting that full reconstitution of the memory B cell pool may require transfer of the different antigen-experienced B cell subsets. Importantly, we found that the recall responses were more efficient if the transferred memory cells were given the required time to adapt to their new environment, suggesting that a period of accommodation is necessary before the transferred cells are fully capable to respond. Our findings also show that different processes can modify the survival conditions of memory B cells. Finally, we found that the generation of the memory B cell pool in response to an optimal dose of Ag did not rely on the number of the initially responding B cells, suggesting autonomous homeostatic controls for naïve and memory B cells a property that may allow reconstitution of the memory pool in immune-deficient hosts using a limited number of precursor naïve B cells. An autonomous control of the memory B cell pool where each antigenic exposure adds new competing clones supports the notion of vaccination strategies using antigenic boosting to prevent memory B cell attrition. Overall the findings reported demonstrate that it is possible to reconstitute the memory B cell pool of an immune deficient host with an artificially induced population of monoclonal high affinity memory B cells.
